# Risk Assessment of Non‐Urinary Tract Recurrence After Radical Nephroureterectomy Based on CheckMate 274 Trial Eligibility: A Multicenter Retrospective Study

**DOI:** 10.1111/iju.70497

**Published:** 2026-05-08

**Authors:** Tetsuya Shindo, Yohei Ueki, Ippei Muranaka, Genki Kobayashi, Shintaro Miyamoto, Yasuharu Kunishima, Shunsuke Sato, Manabu Okada, Shuichi Kato, Ryuichi Kato, Hideki Adachi, Masanori Matsukawa, Akio Takayanagi, Kosuke Shibamori, Atsushi Wanifuchi, Yuki Kyoda, Kohei Hashimoto, Ko Kobayashi, Toshiaki Tanaka, Naoya Masumori

**Affiliations:** ^1^ Department of Urology Sapporo Medical University School of Medicine Sapporo Japan; ^2^ Department of Urology Japan Community Health Care Organization Hokkaido Hospital Sapporo Japan; ^3^ Department of Urology Japanese Red Cross Kushiro Hospital Kushiro Japan; ^4^ Department of Urology Hakodate Goryoukaku Hospital Hakodate Japan; ^5^ Department of Urology Japanese Red Cross Asahikawa Hospital Asahikawa Japan; ^6^ Department of Urology Sunagawa City Medical Center Sunagawa Japan; ^7^ Department of Urology Oji General Hospital Tomakomai Japan; ^8^ Department of Urology Hokkaido Social Work Association Obihiro Hospital Obihiro Japan; ^9^ Department of Urology Steel Memorial Muroran Hospital Muroran Japan; ^10^ Department of Urology Muroran City General Hospital Muroran Japan; ^11^ Department of Urology Saiseikai Otaru Hospital Otaru Japan; ^12^ Department of Urology Takikawa Municipal Hospital Takikawa Japan; ^13^ Department of Urology NTT East Medical Center Sapporo Sapporo Japan

**Keywords:** adjuvant therapy, CheckMate 274 criteria, recurrence, untreated, upper urinary tract carcinoma

## Abstract

**Objectives:**

To assess the risk of non‐urinary tract recurrence after radical nephroureterectomy for patients with upper urinary tract carcinoma according to CheckMate 274 trial eligibility.

**Methods:**

We evaluated patients who underwent radical nephroureterectomy between 2012 and 2022 for upper urinary tract carcinoma. Recurrence was defined as extra‐urinary tract recurrence. Univariate and multivariable analyses were used to determine the risk factors for recurrence according to CheckMate 274 trial eligibility.

**Results:**

Among 545 patients, 127 (23.3%) experienced recurrence during the follow‐up period. Three‐hundred sixty two (66.4%) patients were not eligible for CheckMate 274 trial criteria while 183 (33.6%) patients were eligible. The multivariable analysis demonstrated that preoperative renal dysfunction (HR2.24, 95% CI: 1.37–3.65, *p* = 0.001), presence of lymphovascular invasion (HR1.89, 95% CI: 1.18–3.00, *p* = 0.008)., positive resection margin (HR2.58, 95% CI: 1.43–4.66, *p* = 0.002), positive lymph node status (HR: 2.34, 95% CI: 1.21–4.55, *p* = 0.012) were risk factors for CheckMate 274 eligible patients. On the other hand, among 362 ineligible patients, performing preoperative diagnostic ureteroscopy (HR 3.09, 95% CI: 1.55–6.17, *p* = 0.001), presence of lymphovascular invasion (HR3.81, 95% CI: 1.78–8.17, *p* < 0.001), presence of histological subtype (HR4.82, 95% CI: 1.91–12.13, *p* < 0.001) and pathological T2 stage (HR2.28, 95% CI: 1.08–4.81, *p* = 0.031) were risk factors for recurrence.

**Conclusions:**

According to our study, it may be possible to discuss the needs of adjuvant treatment in each individual. These findings provide a basis for more informed decisions regarding adjuvant therapy in UTUC, with the potential to reduce unnecessary treatment.

## 1 Introduction

Upper urinary tract carcinoma (UTUC) is a malignancy that arises from the renal pelvis and ureter. Surgical approaches are typically recommended for localized UTUC, including radical nephroureterectomy or partial ureterectomy [1, 2]. Over the past decades, surgical techniques have evolved from open surgery to laparoscopic and robot‐assisted approaches [3, 4, 5]. Despite these advancements, survival outcomes have not significantly improved [6]. Therefore, effective perioperative treatment strategies are urgently needed to enhance prognosis in this field.

Neoadjuvant chemotherapy has been reported to improve survival in patients with UTUC [7, 8, 9]. Given that renal dysfunction is often unavoidable following radical nephroureterectomy, administering chemotherapy preoperatively—particularly cisplatin‐based regimens—is considered reasonable. However, no phase III randomized controlled trials have yet demonstrated a definitive survival benefit of neoadjuvant chemotherapy in UTUC patients. Consequently, neoadjuvant therapy is not routinely adopted in UTUC compared to muscle‐invasive bladder cancer (MIBC), for which level I evidence supports its use [10].

Recently, the CheckMate 274 trial demonstrated a survival benefit of adjuvant nivolumab in patients with muscle‐invasive urothelial carcinoma, including both bladder cancer and UTUC, following radical surgery [11, 12, 13]. In this trial, eligible pathological stages for randomization were pT3–4 N0 or pTanyN1 in patients who had not received neoadjuvant chemotherapy (NAC), and ypT2–4 N0 or ypTanyN1 in those who had undergone NAC. However, subgroup analyses raised concerns regarding the lack of survival benefit specifically in UTUC patients [13]. Additionally, immune‐related adverse events associated with immune checkpoint inhibitors remain a clinical challenge [14, 15].

Therefore, it is essential to stratify recurrence risk among patients eligible for CheckMate 274 to avoid both overtreatment and undertreatment. Furthermore, according to the POUT trial, pT2 patients are also candidates for adjuvant chemotherapy [16]. Thus, we propose risk stratification for recurrence even in patients not eligible for CheckMate 274, aiming to identify subpopulations that may derive significant benefit from adjuvant chemotherapy following radical nephroureterectomy.

## 2 Materials and Methods

### 2.1 Study Population and Design

Clinical and pathological data of patients who underwent radical nephroureterectomy (RNU) and were pathologically diagnosed with urothelial carcinoma (UC) between January 2012 and December 2022 were retrospectively collected from 14 participating hospitals. Patients were excluded if they had a follow‐up period of less than 3 months, a history of or concurrent radical cystectomy, clinically positive lymph node status, or had received adjuvant therapy. Moreover, patients whose final pathology revealed renal cell carcinoma despite preoperative suspicion of UTUC were excluded. Urothelial carcinoma with variant histology (e.g., squamous or glandular differentiation) was included in the analysis (Figure S1).

Radiographic evaluations using computed tomography were performed every 3 months during the first 2 years following RNU, every 6 months thereafter, and every 6 to 12 months beyond 5 years postoperatively. Recurrence in this study was defined as extra‐urinary tract tumor and non‐urinary tract recurrence was defined as recurrence outside the urinary tract, including visceral metastases, retroperitoneal recurrence, and lymph node metastasis. All recurrences were primarily diagnosed radiographically, and pathological confirmation was not routinely required. The time to recurrence was calculated from the date of RNU to the first radiographically or pathologically confirmed extra‐urinary tract recurrence.

Clinicopathological data extracted from medical records included patient demographics such as gender, age at RNU, Eastern Cooperative Oncology Group performance status (ECOG‐PS), smoking status, body mass index (BMI), and medical history. As tumor characteristics, pathological T and N status, histological subtype, lymphovascular invasion (LVI), resection margin (RM), presence of hydronephrosis, macrohematuria, and as preoperative factors, renal function, use of neoadjuvant therapy, preoperative diagnostic ureteroscopy were extracted. The threshold for estimated glomerular filtration rate (eGFR) was determined using a previous report for cisplatin eligibility [17]. This study was approved by the Institutional Review Board of Sapporo Medical University School of Medicine (approval number 362–1041) and by the ethical committees of all participating institutions.

### 2.2 Endpoints and Statistical Analyses

The primary endpoint of this study was to identify risk factors for extra‐urinary tract recurrence in patients who underwent RNU for UTUC. Patients were stratified according to the eligibility criteria of the CheckMate 274 trial. Specifically, those who had received NAC were considered eligible if they exhibited either ypT2–4 with any nodal status (Nany) or any primary tumor stage (ypTany) with nodal metastasis (N+). Conversely, patients who had not received NAC were deemed eligible if they had pT3–4 with any nodal status or pTany with nodal metastasis. All remaining patients were classified as non‐eligible. Statistical comparisons between groups were performed using the Mann–Whitney U test and chi‐square test for categorical variables, and Student's *t*‐test for continuous variables. A two‐sided *p*‐value of less than 0.05 was considered statistically significant. Non‐urinary tract recurrence‐free survival (NUTRFS) rates were analyzed, and subgroup comparisons were conducted using the log‐rank test. To identify independent risk factors for recurrence, cox proportional hazard analyses were performed using pre‐RNU clinical and pathological variables. The “number of risk factors” was defined as the number of independent predictors identified in the multivariate analysis that were present in each patient.

All statistical analyses were conducted using EZR version 1.35 (Saitama Medical Center, Jichi Medical University).

## 3 Results

Clinical data from 745 patients who underwent RNU were collected from 14 hospitals. After applying the exclusion criteria, 545 patients were included finally. The median follow‐up period was 2.92 years (interquartile range [IQR], 1.04–5.39), during which 127 patients experienced extra‐urinary tract recurrence.

Clinicopathological characteristics stratified by eligibility for the CheckMate 274 trial are summarized in Table 1 Among the 183 patients who met the CheckMate 274 eligibility criteria, the pathological stages were as follows: ypT2N0 (*n* = 3), ypT3N0 (*n* = 14), ypT4N0 (*n* = 3), ypT3N+ (*n* = 2), pT1N+ (*n* = 2), pT2N+ (*n* = 1), pT3N+ (*n* = 8), pT3N0 (*n* = 145), pT4N0 (*n* = 3), and pT4N+ (*n* = 2). In the CheckMate 274–eligible cohort, multivariable analysis identified the following risk factors for extra‐urinary tract recurrence: preoperative estimated glomerular filtration rate (eGFR) < 50 mL/min (hazard ratio [HR], 2.24; 95% confidence interval [CI], 1.37–3.65; *p* = 0.001), presence of lymphovascular invasion (LVI) (HR, 1.89; 95% CI, 1.18–3.00; *p* = 0.008), positive resection margin (RM) (HR, 2.58; 95% CI, 1.43–4.66; *p* = 0.002), and pathological lymph node positivity (HR, 2.34; 95% CI, 1.21–4.55; *p* = 0.012), as shown in Table 2.

**TABLE 1:** Comparison of patient characteristics based on CheckMate 274 eligibility criteria.

	CM 274 ineligible	CM 274 eligible	*P*
	Patients (*n* = 362)	Patients (*n* = 183)	
Median age, (IQR) years	74 (68–79)	77 (71–81)	< 0.001
ECOG‐PS			
PS0	286 (79.0%)	136 (74.3%)	0.233
PS1 or more	76 (21.0%)	47 (25.7%)	
Gender			
Female	103 (28.5%)	66 (36.1%)	0.078
Male	259 (71.5%)	117 (63.9%)	
BMI			
more than 22	117 (32.3%)	69 (37.7%)	0.215
22 or less	245 (67.7%)	114 (62.3%)	
Smoking history			
Yes	248 (69.5%)	114 (63.0%)	0.145
No	109 (30.5%)	67 (37.0%)	
NAC use			
Yes	24 (6.6%)	22 (12.0%)	0.049
No	338 (93.4%)	161 (88.0%)	
DM			
Yes	98 (27.1%)	44 (24.0%)	0.471
No	264 (72.9%)	139 (76.0%)	
Hypertension			
Yes	203 (56.1%)	111 (60.7%)	0.314
No	159 (43.9%)	72 (39.3%)	
Hydronephrosis			
Yes	155 (42.8%)	101 (55.2%)	0.070
No	207 (57.2%)	82 (44.8%)	
eGFR less than 50 ml/min/1.73^2^			
Yes	124 (34.3%)	108 (59.0%)	< 0.001
No	238 (65.7%)	75 (41.0%)	
History of NMIBC			
Yes	107 (29.6%)	29 (15.8%)	0.001
No	255 (70.4%)	154 (84.2%)	
Pathological T stage			
pT1 or less	276 (76.2%)	2 (1.1%)	< 0.001
pT2	86 (23.8%)	4 (2.2%)	
pT3	0 (0.0%)	169 (92.3%)	
pT4	0 (0.0%)	8 (4.4%)	
Pathological *N* stage			
pN (+)	0 (0.0%)	15 (8.2%)	< 0.001
pN (−)	101 (27.9%)	65 (35.5%)	
pNx	261 (72.1%)	103 (56.3%)	
LVI status			
Positive	47 (13.0%)	107 (58.5%)	< 0.001
Negative	315 (87.0%)	76 (41.5%)	
Histological subtype			
Yes	17 (4.7%)	24 (13.1%)	0.001
No	344 (95.3%)	159 (86.9%)	
Resection margin status			
Positive	4 (1.1%)	17 (9.3%)	< 0.001
Negative	358 (98.9%)	166 (90.7%)	

*Note:* Age: Mann–Whitney U test. Categorical variables: chi‑square test; Fisher's exact test when expected cell count less than 5.

Abbreviations: ECOG‐PS, Eastern Cooperative Oncology Group Performance Status; LVI, ymphovascular invasion; NAC, Neoadjuvant chemotherapy; VH, Variant histology.

**TABLE 2:** Uni‐, and multivariate analysis for extra‐urinary tract recurrence free survival according to eligibility of CheckMate 274 criteria.

	CheckMate 274 ineligible (*N* = 362)				CheckMate 274 eligible (*N* = 183)			
	Univariate		Multivariate		Univariate		Multivariate	
	HR (95% CI)	*P*	HR (95% CI)	*P*	HR (95% CI)	*P*	HR (95% CI)	P
Sex (Male)	1.84 (0.85–3.98)	0.123			0.91 (0.59–1.42)	0.685		
ECOG‐PS (1 or more)	1.37 (0.67–2.79)	0.388			1.05 (0.64–1.72)	0.848		
BMI (22 or less)	1.33 (0.71–2.52)	0.376			0.91 (0.59–1.42)	0.669		
Smoking history (Yes)	0.95 (0.49–1.84)	0.890			1.00 (0.64–1.56)	0.988		
History of diabetes mellitus (Yes)	0.87 (0.43–1.78)	0.707			1.40 (0.88–2.24)	0.158		
History of hypertension (Yes)	1.15 (0.62–2.14)	0.665			0.99 (0.64–1.52)	0.949		
Preoperative eGFR (less than 50 ml/min)	1.65 (0.89–3.05)	0.114			2.51 (1.56–4.06)	< 0.001	2.24 (1.37–3.65)	0.001
Hydronephrosis (Yes)	0.94 (0.50–1.75)	0.844			1.47 (0.95–2.27)	0.084		
Preoperative Ureteroscopy (Yes)	2.15 (1.11–4.15)	0.023	3.09 (1.55–6.17)	0.001	0.88 (0.58–1.36)	0.578		
NAC usage (Yes)	0.36 (0.05–2.63)	0.315			1.27 (0.70–2.29)	0.432		
History of NMIBC prior to RNU (Yes)	1.29 (0.68–2.47)	0.437			0.89 (0.50–1.58)	0.691		
Macrohematuria (Yes)	1.14 (0.59–2.21)	0.692			1.23 (0.80–1.90)	0.349		
LVI (Yes)	4.63 (2.45–8.74)	< 0.001	3.81 (1.78–8.17)	< 0.001	2.04 (1.29–3.25)	0.002	1.89 (1.18–3.00)	0.008
High age (75 or older)	1.45 (0.78–2.70)	0.236			1.38 (0.87–2.18)	0.166		
RM positive (Yes)	2.36 (0.32–17.17)	0.398			3.69 (2.07–6.59)	< 0.001	2.58 (1.43–4.66)	0.002
Variant histology (Yes)	4.59 (1.92–10.98)	< 0.001	4.82 (1.91–12.13)	< 0.001	0.66 (0.32–1.36)	0.255		
pT2 (Yes)	4.44 (2.40–8.22)	< 0.001	2.28 (1.08–4.81)	0.031	N/A	N/A		
LND (Yes)	0.86 (0.42–1.77)	0.689			1.32 (0.86–2.04)	0.207		
pN positive (Yes)	N/A	N/A			2.41 (1.24–4.67)	0.009	2.34 (1.21–4.55)	0.012

**FIGURE 1:**
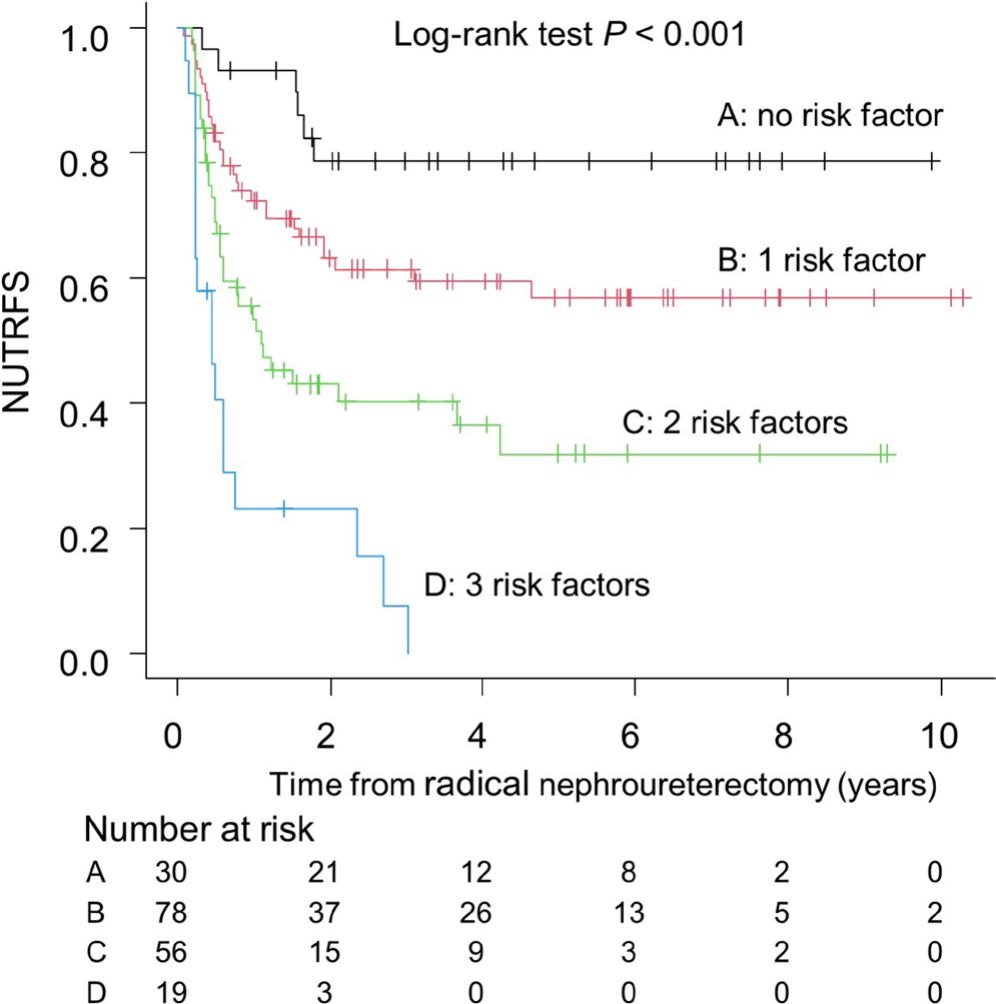
NUTRFS stratified by risk factor count in 183 CheckMate 274–eligible patients.

**FIGURE 2:**
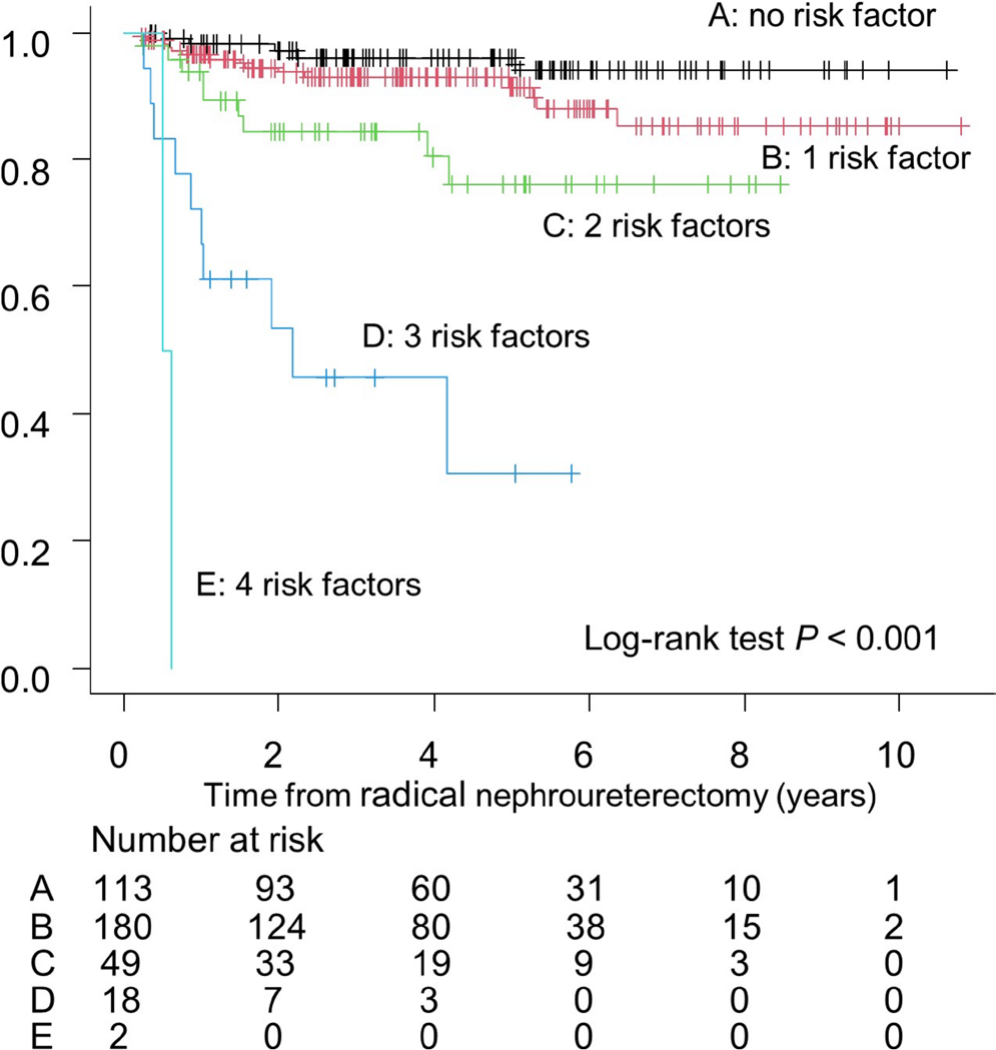
NUTRFS stratified by risk factor count in 362 CheckMate 274–ineligible patients.

Non‐urinary tract recurrence‐free survival (NUTRFS) rates in CheckMate 274 eligible patients were significantly stratified according to the number of risk factors (Figure 1) The 1‐year NUTRFS rates were 93.3%, 72.5%, 53.6%, and 23.2% in patients with 0, 1, 2, and 3 risk factors, respectively (*p* < 0.001, log‐rank test). In contrast, among patients who did not meet the CheckMate 274 eligibility criteria, the following factors were significantly associated with recurrence: performance of preoperative diagnostic ureteroscopy (HR, 3.09; 95% CI, 1.55–6.17; *p* = 0.001), presence of LVI (HR, 3.81; 95% CI, 1.78–8.17; *p* < 0.001), presence of histological subtype (HR, 4.82; 95% CI, 1.91–12.13; *p* < 0.001), and pathological T2 stage (HR, 2.28; 95% CI, 1.08–4.81; *p* = 0.031). The 1‐year NUTRFS rates in this cohort were 98.2%, 96.5%, 93.7%, 72.2%, and 0% in patients with 0, 1, 2, 3, and 4 risk factors, respectively (*p* < 0.001, log‐rank test; Figure 2). The predictive factors for extra‐urinary tract recurrence in the entire 545 patients were demonstrated in supplementary Table 1 The distribution of specific risk factors or combinations according to the number of risk factors (risk number 1–3) in CheckMate 274 ineligible and eligible populations is demonstrated in supplementary Figures 2 and 3.

## 4 Discussion

In this study, we identified risk factors for NUTRFS in UTUC patients who underwent RNU, stratified according to the eligibility criteria of the CheckMate 274 trial. Our findings suggest that relying solely on the CheckMate 274 criteria to determine indications for adjuvant therapy may lead to both undertreatment and overtreatment in clinical practice.

In our CheckMate 274–ineligible cohort, the rate of non‐urinary tract recurrence was significantly higher in patients with 2 or more risk factors compared to those with 0–1 (HR 5.38, 95% CI 2.91–9.95, *p* < 0.001; Cox proportional hazards analysis). Among the 69 patients in the ineligible group who had 2 or more risk factors, the 1‐year NUTRFS was 85.2% which was lower than the 1‐year NUTRFS, 93.3% in CheckMate 274–eligible patients with no risk factors. These findings suggest that a more aggressive approach to adjuvant therapy may be warranted in patients with multiple risk factors, even if they do not meet the formal eligibility criteria of the CheckMate 274 trial.

Indeed, there is an issue that we could not apply adjuvant nivolumab therapy because of patient selection criteria based on pathological T and N status. However, adjuvant chemotherapy strategies which is supported by POUT trial is suitable if the patient had pT2 status. In our CheckMate 274–ineligible cohort, 69 patients had 2 or more risk factors. Among them, only 4 patients had pT1 disease, indicating that 65 of the 69 (94.2%) patients met the criteria for adjuvant chemotherapy based on the POUT trial. On the contrary, in CheckMate 274 eligible patients, 30 of 183 (16.4%) patients had none of the demonstrated risk factors. Among this subgroup, as mentioned, 1‐year NUTRFS was 93.3%. According to the updated CheckMate 274 trial report, any‐grade and grade 3 to 4 adverse events occurred 78.6% and 18.2%, respectively [11]. Therefore, it may be justified to avoid adjuvant nivolumab treatment to reduce the risk of overtreatment and possible immune related adverse events. Nonetheless, multivariable analysis identified eligibility for the CheckMate 274 criteria as a risk factor for NUTRFS in the overall cohort of 545 patients. This finding implies that meeting the CheckMate 274 criteria alone may confer a certain level of risk. Therefore, the indication for adjuvant therapy should be carefully considered on a case‐by‐case basis. In particular, we believe that the risk of being ineligible for systemic therapy at the time of recurrence after surgery for upper tract urothelial carcinoma, as we previously reported [18], should also be taken into account.

A survival benefit was demonstrated in the CheckMate 274 trial with 1 year of adjuvant nivolumab following radical surgery for muscle‐invasive urothelial carcinoma, including both bladder cancer and UTUC [13]. However, such survival benefit is not clearly established when limited to UTUC patients. Otiato M et al. reported non‐superiority of adjuvant immunotherapy after RNU for UTUC patients [19]. However, this study included several immunotherapy agents such as nivolumab, pembrolizumab, and atezolizumab. More precisely, only 11 patients were treated with adjuvant nivolumab. Additional real‐world data are needed to clarify the oncological benefit of adjuvant nivolumab specifically in UTUC. From another perspective, given the current uncertainty regarding its efficacy, efforts should be made to avoid overtreatment.

The risk factors for recurrence in the CheckMate 274–ineligible cohort included preoperative ureteroscopy, presence of LVI, histological variants, and pathological T2 status. Preoperative ureteroscopy is a well‐established risk factor for intravesical recurrence. However, this procedure has not been previously reported as a risk factor for systemic recurrence or metastasis, which contrasts with our findings. This discrepancy may suggest that ureteroscopy primarily influences recurrence in patients with low pathological T stage. Although this mechanism has not been directly validated in UTUC, evidence from bladder cancer suggests a plausible explanation. In TURBT, elevated intravesical irrigation pressure has been reported to facilitate the entry of tumor cells into the bloodstream, as shown by studies demonstrating an increase in circulating tumor cells immediately after the procedure [20]. These findings imply that endoscopic manipulation under continuous irrigation can promote intraoperative tumor cell dissemination. While definitive evidence supporting the same mechanism in ureteroscopy is lacking, it is reasonable to assume that irrigation pressure may rise during ureteroscopy in certain cases, potentially allowing tumor cells to escape the urinary tract and contribute to extraurothelial recurrence. In patients who met the CheckMate 274 high risk criteria, factors intrinsic to tumor biology, such as lymphovascular invasion, may exert a dominant influence on recurrence risk, thereby attenuating or masking any incremental effect of ureteroscopy. This pattern suggests that ureteroscopy related microdissemination may become clinically apparent primarily in lower risk patients, whereas in high risk disease, tumor driven factors overshadow the procedural impact. From another perspective, we should be cautious about performing preoperative ureteroscopy. We identified 69 patients within the CheckMate 274‐ineligible cohort of 362 cases who had two, three, or four recurrence risk factors. Notably, 49 of these patients (71.0%) had preoperative ureteroscopy included among their risk‐factor combinations (Figure 2). Because the other components in this group, VH positivity, LVI, and ≥ pT2 disease, are intrinsic tumor characteristics, this finding suggests that avoiding ureteroscopic manipulation may help reduce the risk of extraurothelial recurrence when UTUC is already evident on imaging or urine cytology. The decision to perform or omit preoperative ureteroscopy was based on clinical judgment, and this may have introduced indication bias. Although tumor information was limited, we examined tumor multiplicity and tumor location according to diagnostic ureteroscopy status. Multiple tumors and ureteral tumors were slightly more frequent in the preoperative ureteroscopy group; however, neither characteristic was a risk factor for non–urinary tract recurrence in the CheckMate 274‐ineligible cohort (data not shown). In contrast, preoperative ureteroscopy remained an independent risk factor in our analysis, which we consider an important finding of this study. Since no previous studies have evaluated the impact of ureteroscopy on non‐urinary tract recurrence following RNU in non‐advanced UTUC population, this topic warrants further investigation using larger cohorts.

Histological variants also emerged as a recurrence risk factor in the ineligible cohort, but not in the eligible group. Jeon et al. [21] reported that adjuvant chemotherapy may improve oncological outcomes in patients with UTUC who exhibit histological variants. Therefore, even patients who do not meet the eligibility criteria for the CheckMate 274 trial may still be potential candidates for adjuvant chemotherapy if histological variants are present. LVI was the only risk factor which was common in both eligible and ineligible cohorts. There were many previous studies reporting impact of LVI on worse oncological outcomes [22, 23, 24, 25]. Zhang et al. reported that LVI correlated to worse recurrence free survival, CSS and OS in UTUC patients after surgery using meta‐analysis [22]. LVI is typically associated with higher pathological stages; therefore, reports on its impact in low pathological T stages are rare. Kagawa et al. reported the clinical significance of LVI in patients with UTUC who underwent RNU [25]. According to their findings, patients who were ineligible for the CheckMate 274 trial and had LVI‐positive disease exhibited poorer recurrence‐free survival compared to those who were eligible but LVI‐negative. Our data also demonstrates the impared renal function was the independent risk factor for NUTRFS in CheckMate 274 eligible patients. Preoperative renal dysfunction has been reported as a risk factor for recurrence in patients with UTUC [26], and various mechanisms have been proposed to explain this association. Among them, β2‐microglobulin (β2MG), a well‐established biomarker of renal tubular injury, has been implicated in recurrence risk [27]. β2MG is also a component of the major histocompatibility complex class I (MHC‐I) molecule and plays a critical role in immune responses [28]. At elevated concentrations, β2MG has been shown to impair the immune microenvironment [29]. Furthermore, it may promote tumor progression by enhancing the production of pro‐inflammatory cytokines, thereby altering the tumor microenvironment in a manner conducive to recurrence and metastasis. In addition, chronic kidney disease (CKD) has been associated with pan‐urothelial cancerization due to frequent exposure to nephrotoxic agents, systemic inflammation, and uremia‐induced immune dysfunction, including impaired T cell activation [30]. These factors may contribute to carcinogenesis and tumor progression in UTUC, highlighting the multifactorial nature of the relationship between renal impairment and poor oncologic outcomes. In our study, the threshold for eGFR was determined using ROC curve analysis. Notably, when treated as a continuous variable, eGFR remained a statistically significant predictor of extra–urinary tract recurrence in the CheckMate 274–eligible cohort.

This study has several limitations. First, only 46 of 545 (8.4%) of patients in our cohort received NAC. Although level 1 evidence supporting NAC for UTUC is still lacking, its clinical use is gradually expanding [8, 9]. Therefore, our findings should be interpreted with caution, as they may not fully reflect outcomes in future cohorts with broader NAC implementation. Second, lymph node dissection was omitted in 364 of 545 (66.8%) patients at the time of RNU. This variability in lymph node dissection may have introduced selection bias. In this cohort, the decision to perform lymph node dissection depended on the surgeon's judgment, potentially reflecting preoperative assessments of tumor aggressiveness and functioning as a source of confounding by indication. In our multivariable cox model including lymph node dissection status as a covariate, lymph node dissection was not associated with extraurothelial recurrence.

However, this heterogeneity in surgical practice may introduce hidden bias, potentially affecting oncological outcomes and limiting the generalizability of our results. Third, we proposed a novel concept for guiding adjuvant therapy decisions in both CheckMate 274–eligible and –ineligible populations. However, our current data do not demonstrate a definitive benefit of adjuvant therapy in reducing non–urinary tract recurrence. Prospective real‐world studies will be necessary to validate this approach and clarify its clinical utility.

In conclusion, we demonstrated a risk stratification framework for non–urinary tract recurrence following RNU, based on the eligibility criteria of the CheckMate 274 trial. Our risk stratification model is intended to characterize perioperative factors associated with non–urinary tract recurrence and to support clinical discussion. These findings should not be interpreted as direct recommendations regarding the use or omission of adjuvant therapy, as our study did not evaluate adjuvant treatment strategies. Prospective validation will be required before this stratification can be applied to guide therapeutic decision making.

## Author Contributions

**Genki Kobayashi:** data curation. **Yohei Ueki:** data curation, investigation. **Tetsuya Shindo:** conceptualization, methodology, data curation, investigation, formal analysis, supervision, project administration, visualization, funding acquisition, writing – original draft, writing – review and editing, validation, software, resources. **Ippei Muranaka:** data curation. **Hideki Adachi:** data curation. **Shintaro Miyamoto:** data curation. **Masanori Matsukawa:** data curation. **Ryuichi Kato:** data curation. **Shunsuke Sato:** data curation. **Manabu Okada:** data curation. **Akio Takayanagi:** data curation. **Yasuharu Kunishima:** data curation. **Atsushi Wanifuchi:** data curation. **Yuki Kyoda:** methodology, conceptualization. **Shuichi Kato:** data curation. **Toshiaki Tanaka:** conceptualization, methodology. **Kohei Hashimoto:** conceptualization, methodology. **Naoya Masumori:** conceptualization, methodology, writing – original draft, writing – review and editing, supervision. **Kosuke Shibamori:** data curation. **Ko Kobayashi:** conceptualization, methodology.

## Disclosure

Approval of the research protocol by an Institutional Reviewer Board: The study was approved by the Ethical Committee of Sapporo Medical University School of Medicine (institutional review board number 362–1041) and the ethical committees of each participating institution.

## Consent

This study was conducted using an opt‐out methodology. Participants were informed about the study and were given the opportunity to opt out if they did not wish to participate. Therefore, individual informed consent was not required.

## Conflicts of Interest

Naoya Masumori, Editor‐in‐Chief, and Kohei Hashimoto, Managing Editor of the International Journal of Urology, are co‐authors of this article. They were excluded from all editorial decisions regarding the acceptance and publication of this manuscript.

Ko Kobayashi is an Editorial Board member of International Journal of Urology and a co‐author of this article. To minimize bias, they were excluded from all editorial decision‐making related to the acceptance of this article for publication.

## Supporting information


**Figure S1:** Flow diagram showing exclusion criteria and final study population.
**Figure S2:** Distribution of specific risk factor or combinations according to the number of risk factors (risk number 1–3) in CM274 ineligible population.
**Figure S3:** Distribution of specific risk factor or combinations according to the number of risk factors (risk number 1–3) in CM274 eligible population.

**Table S1** Uni‐, and multivariable analysis for extra‐urinary tract recurrence free survival among entire patients.

## Data Availability

The data that support the findings of this study are available from the corresponding author upon reasonable request.
