# Supplementary material for: Risk Assessment of Non‐Urinary Tract Recurrence After Radical Nephroureterectomy Based on CheckMate 274 Trial Eligibility: A Multicenter Retrospective Study
Source: Int J Urol. 2026 May 8;33:e70497. doi: 10.1111/iju.70497 (PMC13156526; doi:10.1111/iju.70497)
Supplement: Supplementary file 1 — Figure S1: Flow diagram showing exclusion criteria and final study population. Figure S2: Distribution of specific risk factor or combinations according to the number of risk factors (risk number 1–3) in CM274 ineligible population. Figure S3: Distribution of specific risk factor or combinations according to the number of risk factors (risk number 1–3) in CM274 eligible population. [file IJU-33-0-s002.pptx]

## Slide 1
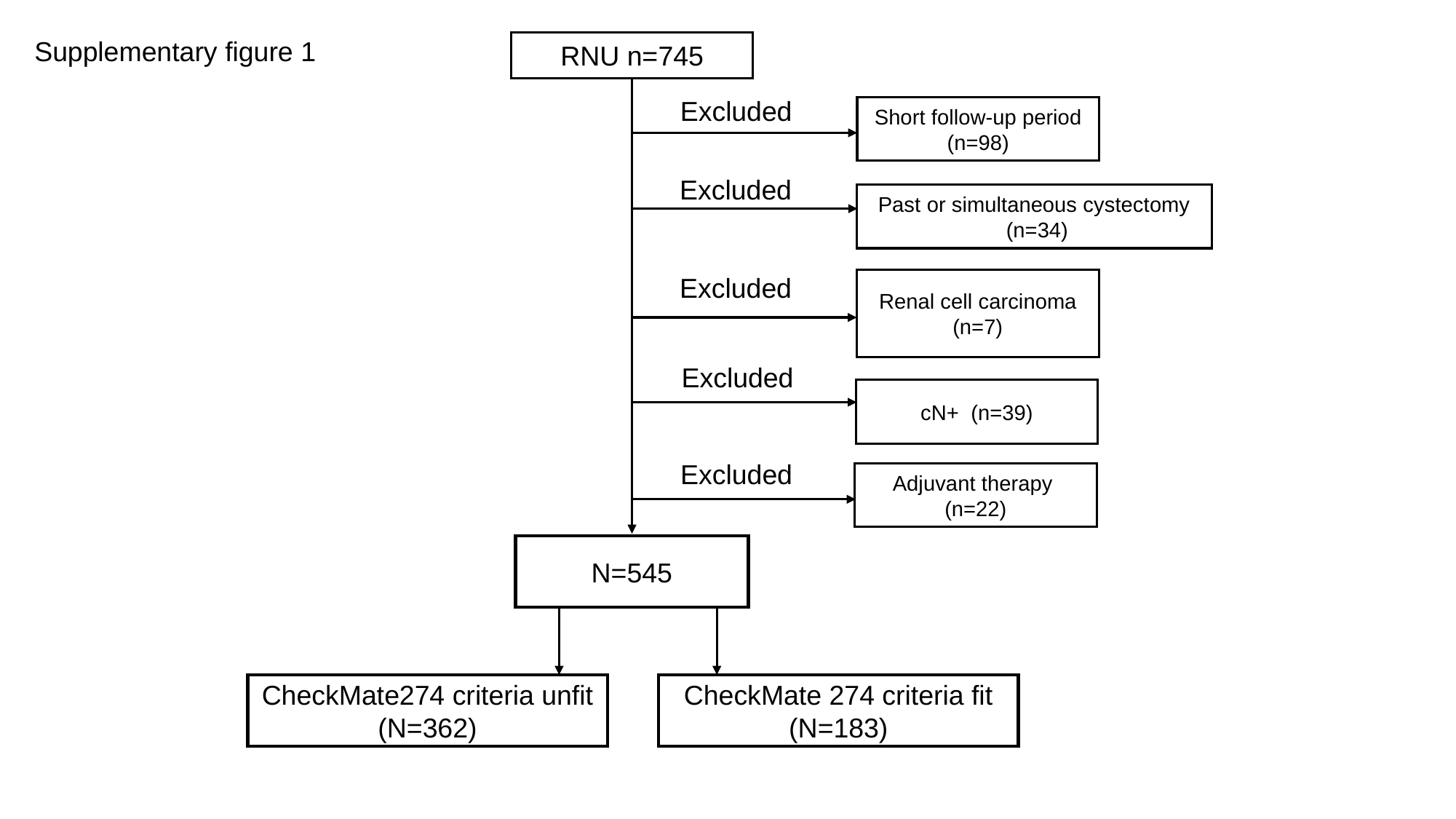

Supplementary figure 1
RNU n=745
Excluded
Short follow-up period (n=98)
Excluded
Past or simultaneous cystectomy
 (n=34)
Excluded
Renal cell carcinoma (n=7)
Excluded
cN+ (n=39)
Excluded
Adjuvant therapy (n=22)
N=545
CheckMate274 criteria unfit (N=362)
CheckMate 274 criteria fit (N=183)

## Slide 2
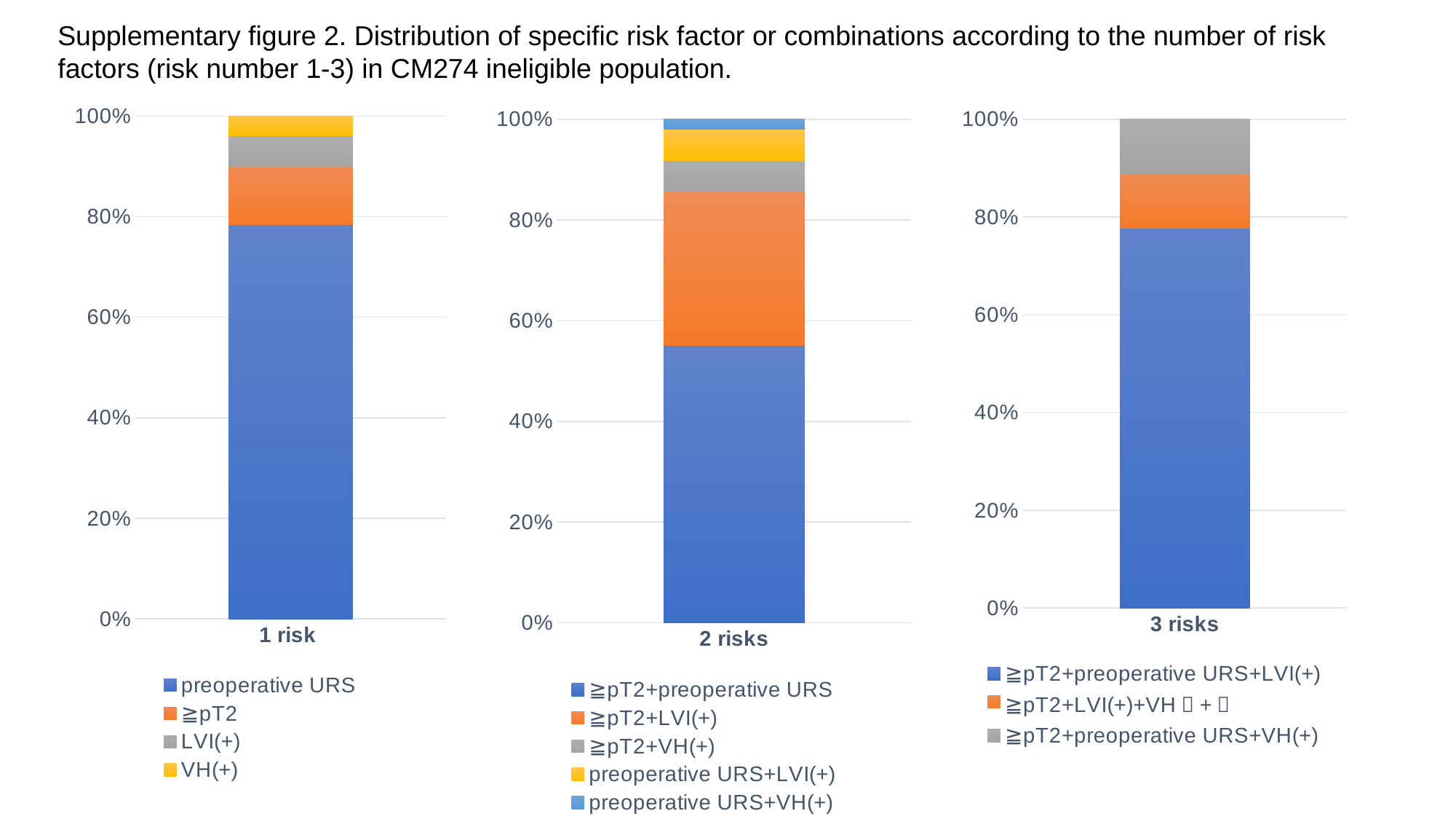

Supplementary figure 2. Distribution of specific risk factor or combinations according to the number of risk factors (risk number 1-3) in CM274 ineligible population.
### Chart
| Category | preoperative URS | ≧pT2 | LVI(+) | VH(+) |
|---|---|---|---|---|
| 1 risk | 141.0 | 21.0 | 11.0 | 7.0 |
### Chart
| Category | ≧pT2+preoperative URS | ≧pT2+LVI(+) | ≧pT2+VH(+) | preoperative URS+LVI(+) | preoperative URS+VH(+) |
|---|---|---|---|---|---|
| 2 risks | 27.0 | 15.0 | 3.0 | 3.0 | 1.0 |
### Chart
| Category | ≧pT2+preoperative URS+LVI(+) | ≧pT2+LVI(+)+VH（+） | ≧pT2+preoperative URS+VH(+) |
|---|---|---|---|
| 3 risks | 14.0 | 2.0 | 2.0 |

## Slide 3
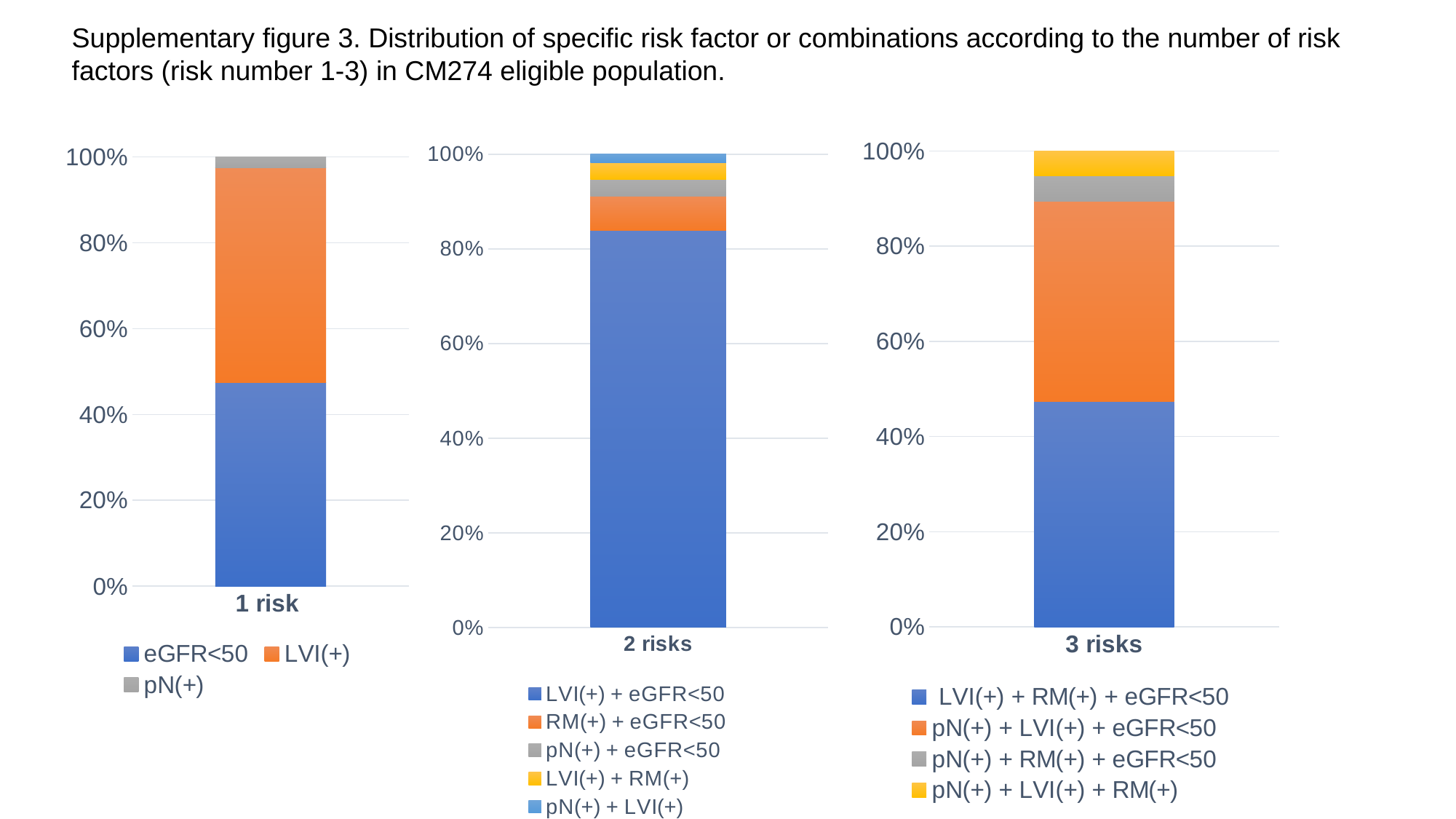

Supplementary figure 3. Distribution of specific risk factor or combinations according to the number of risk factors (risk number 1-3) in CM274 eligible population.
### Chart
| Category | LVI(+) + RM(+) + eGFR<50 | pN(+) + LVI(+) + eGFR<50 | pN(+) + RM(+) + eGFR<50 | pN(+) + LVI(+) + RM(+) |
|---|---|---|---|---|
| 3 risks | 9.0 | 8.0 | 1.0 | 1.0 |
### Chart
| Category | LVI(+) + eGFR<50 | RM(+) + eGFR<50 | pN(+) + eGFR<50 | LVI(+) + RM(+) | pN(+) + LVI(+) |
|---|---|---|---|---|---|
| 2 risks | 47.0 | 4.0 | 2.0 | 2.0 | 1.0 |
### Chart
| Category | eGFR<50 | LVI(+) | pN(+) |
|---|---|---|---|
| 1 risk | 37.0 | 39.0 | 2.0 |
